# Macrotrabecular-Massive Hepatocellular Carcinoma: A Case Report

**DOI:** 10.7759/cureus.75989

**Published:** 2024-12-19

**Authors:** Raphaël Eftimie Spitz, Simona Manole, Teodora Surdea-Blaga, Cosmin Caraiani, Claudia Burz

**Affiliations:** 1 Department of Clinical Immunology and Allergology, Iuliu Hatieganu University of Medicine and Pharmacy of Cluj, Cluj-Napoca, ROU; 2 Department of Radiology and Imaging, Iuliu Hatieganu University of Medicine and Pharmacy of Cluj, Cluj-Napoca, ROU; 3 Department of Internal Medicine, Iuliu Hatieganu University of Medicine and Pharmacy of Cluj, Cluj-Napoca, ROU; 4 Department of Medical Imaging and Nuclear Medicine, Iuliu Hatieganu University of Medicine and Pharmacy of Cluj, Cluj-Napoca, ROU; 5 Department of Medical Oncology, Oncology Institute "Prof. Dr. Ion Chiricuţă" Cluj-Napoca, Cluj-Napoca, ROU

**Keywords:** abdominal ct scans, artificial intelligence, case report, chest ct scan, hbv infection, hepatocarcinoma, immunotherapy, macrotrabecular-massive hepatocellular carcinoma, mri, pet-ct scan

## Abstract

Macrotrabecular-massive hepatocellular carcinoma (MTM-HCC) is a rare and aggressive molecular subtype of hepatocellular carcinoma (HCC) associated with a poor prognosis. Unlike typical HCC, which commonly arises in the context of cirrhosis, MTM-HCC can develop in non-cirrhotic livers, presenting unique diagnostic and therapeutic challenges. This case report describes a 35-year-old male who presented with persistent epigastric pain, fatigue, and loss of appetite. Clinical examination revealed hepatomegaly, prompting advanced imaging and laboratory investigations. Imaging studies identified a large hepatic mass with portal vein thrombosis and metastatic lesions, while histopathological analysis confirmed the diagnosis of MTM-HCC.

The patient initiated treatment with a combination of immune checkpoint inhibitors and anti-angiogenic agents, which represent the current standard for advanced HCC. Despite initial adherence, disease progression was observed after four cycles of therapy. The patient passed away less than two months after his last consultation. This clinical course highlights the aggressive nature of MTM-HCC and its limited responsiveness to existing therapeutic protocols.

MTM-HCC is characterized by distinctive histological and molecular features that differentiate it from other HCC subtypes. These include specific genetic mutations and protein expression patterns that contribute to its aggressive behavior and poor prognosis. Advanced imaging modalities combined with histopathological analysis remain crucial for accurate diagnosis and classification.

This case emphasizes the critical need for heightened clinical vigilance, particularly in younger patients with atypical presentations of liver disease. It also underscores the importance of developing more effective, tailored therapeutic strategies for MTM-HCC. Further research into its molecular characteristics and inclusion in clinical trials is essential to improving outcomes for patients with this challenging and understudied subtype of liver cancer.

## Introduction

Primary liver cancer is responsible for 830,000 deaths worldwide each year [1]. According to GLOBOCAN data, a global increase in incidence was reported, with 906,000 new cases in 2020 [1]. Nonetheless, a declining incidence was observed in the United States [2] and the Asia-Pacific region [3], as noted in the most recent literature to date. It is mainly represented by hepatocellular carcinoma (HCC). HCC risk factors include hepatitis B virus (HBV), hepatitis C virus (HCV), alcohol consumption, or aflatoxin exposure. Eighty percent of the time, these risk factors lead to liver cirrhosis, and HCC occurs as a complication of liver cirrhosis [4]. Nonetheless, HCC rarely develops without liver cirrhosis; this applies to the case in our report.

Recently, a new subtype of HCC entered the classification of different subtypes of HCC in its latest version from 2019, World Health Organization (WHO): the macrotrabecular-massive hepatocellular carcinoma (MTM-HCC) [5]. This subtype of HCC is substantially linked with HBV [6], as it was linked to our case. It is characterized by a distinctive microscopic feature: prominent, thick trabeculae measuring more than 6 cells in thickness, which are present in at least 50% of the tumor [7]. This subtype is notably rare; its prognosis is particularly poor due to the coexpression of CMTM6 (an immune checkpoint inhibitor) and PD-L1 [8]. Since MTM-HCC was first described in 2017 [9], there have been limited studies on its clinical behavior, molecular profile, and optimal treatment strategies. Our report aims to contribute to the growing body of knowledge on this rare subtype, highlighting the importance of early diagnosis and the potential role of emerging therapeutic targets.

## Case presentation

A 35-year-old male presented to our hospital with persistent epigastric pain lasting several months, along with asthenia and loss of appetite. He denied any significant past medical or surgical history. On physical examination, diffuse hepatomegaly was noted, extending approximately 4 cm below the costal margin. It was firm in texture and slightly tender on palpation. Considering the patient's young age, clinicians initially considered rare diseases such as Wilson's disease, granulomatous disease, or parasitic infections. Gastroscopy revealed severe portal hypertensive gastropathy in the stomach body, a common finding in the context of portal hypertension associated with liver failure.

**Figure 1:**
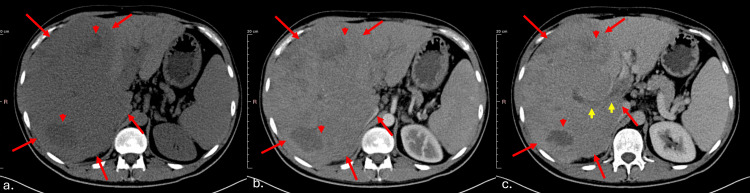
Abdominal CT scan: (a) non-enhancement, (b) arterial phase, and (c) portal phase A voluminous tumoral mass (red arrows) occupying almost the entire right hepatic lobe, with lobulated borders, hypodense appearance on the native phase, inhomogeneous contrast enhancement, and areas of necrosis (red arrowheads). Portal vein thrombosis is also observed (yellow arrows). CT: computed tomography

A contrast-enhanced abdominal computed tomography (CT) scan revealed a voluminous hypodense lesion in the right hepatic lobe, measuring approximately 19 x 15 x 11 centimeters, entirely occupying the right lobe with lobulated borders and extending to the subcapsular region. The lesion showed inhomogeneous contrast enhancement with necrotic areas. Obstructive thrombosis was observed in both the right and left portal branches, appearing hyperdense on contrast imaging (Figure 1a, 1b, 1c). At this stage, the radiologist suggested the differential diagnosis of a primary hepatic lymphoma or infiltrative HCC.

**Figure 2:**
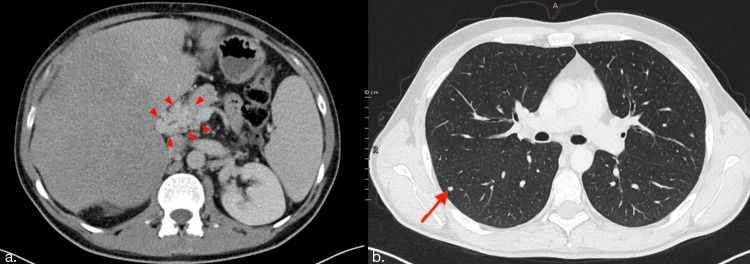
Axial abdominal CT scan venous phase (a) and thoracic CT scan in the pulmonary window (b) a: Peritoneal carcinomatosis is indicated by a red arrowhead. b: A suspicious pulmonary nodule is marked by a red arrow. CT: computed tomography

A complementary thoracic and pelvic CT scan was performed, revealing suspicious pulmonary nodules (Figure 2a) and a suspicious nodule suggestive of peritoneal carcinomatosis on the abdominal CT scan (Figure 2b). The pelvic floor showed no pathological abnormalities.

**Figure 3:**
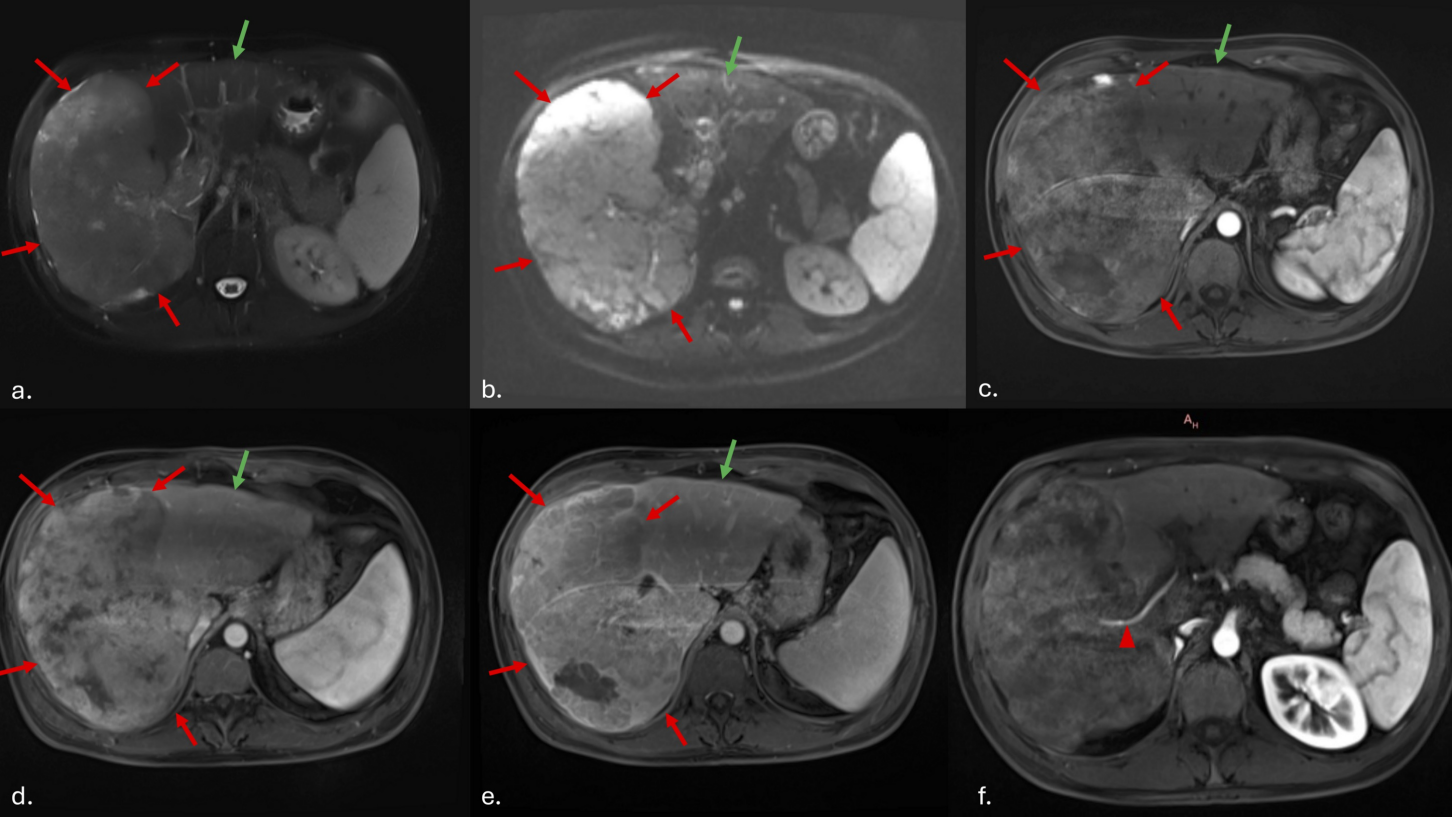
Imaging aspect of a MTM-HCC The lesion shows discrete hypersignal on the T2 weighted image (a) and restricted diffusion (b). Some patchy enhancement is to be seen in the arterial (c) and porto-venous phases (d), with wash-out on the late phase (e). The tumor is indicated by red arrows, the preserved left lobe by a green arrow, and an intratumoral artery—a characteristic ancillary finding of MTM-HCC—by a red arrowhead (f). MTM-HCC: macrotrabecular-massive hepatocellular carcinoma

**Figure 4:**
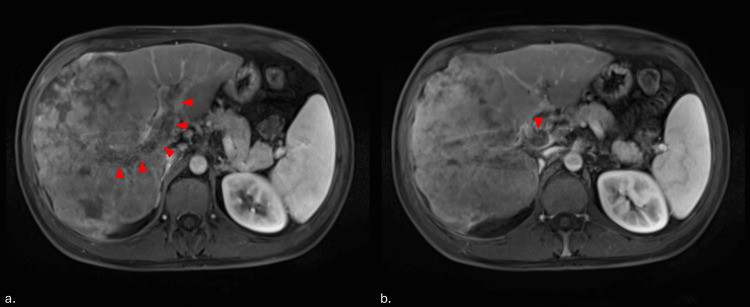
Tumoral portal vein thrombosis a: A tumoral portal vein thrombosis is indicated by a red arrowhead. b: Portal cavernoma, developed in the liver hilum as a consequence of portal vein thrombosis, is marked by a single red arrowhead.

An MRI revealed typical findings consistent with MTM-HCC (Figures 3-4).

**Figure 5:**
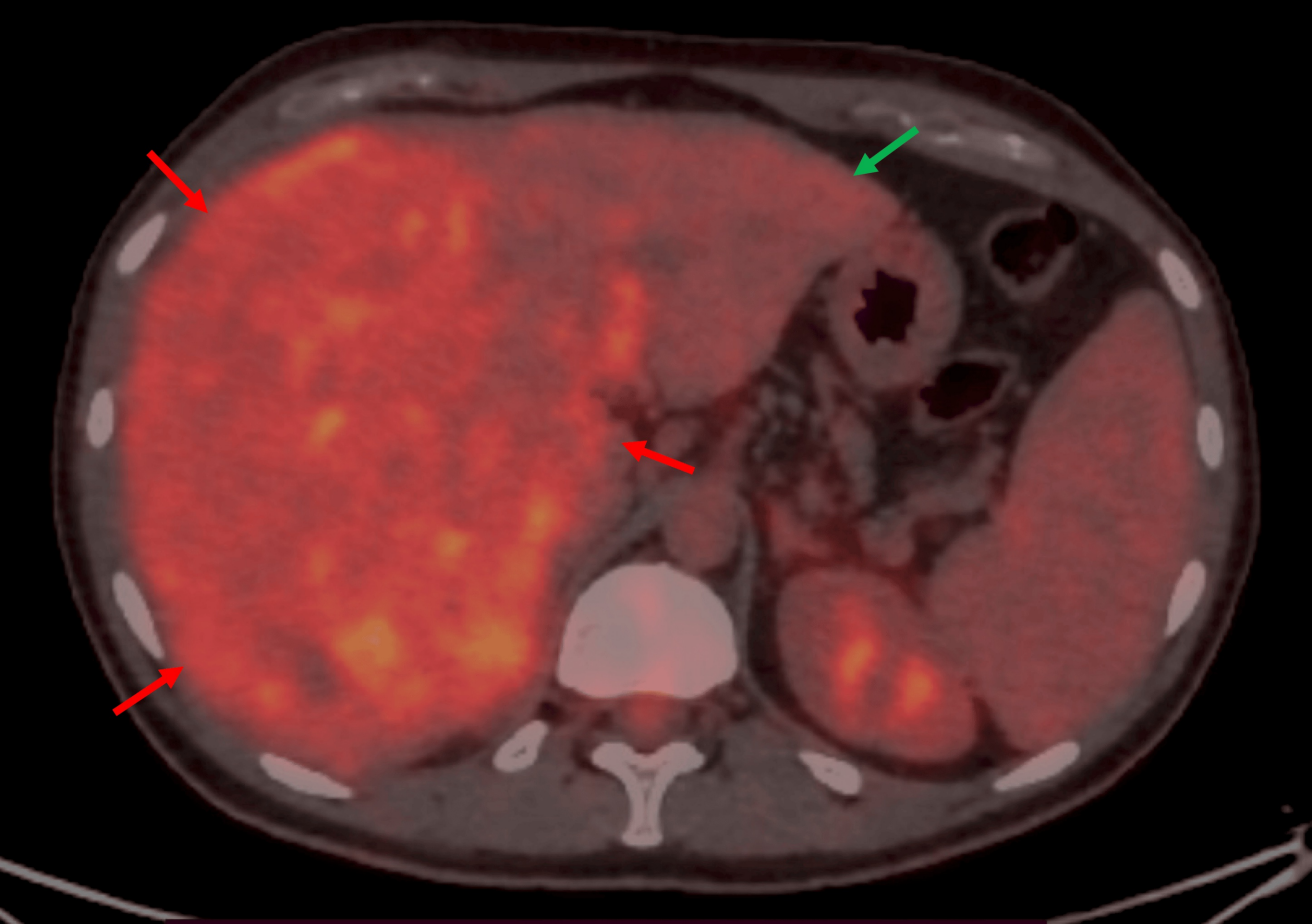
MTM-HCC findings on PET-CT scan Intense, diffuse, and inhomogeneous FDG uptake is observed in the hepatic tumor mass. The tumor is marked by red arrows, and the preserved left lobe is indicated by a green arrow. MTM-HCC: macrotrabecular-massive hepatocellular carcinoma, PET-CT: positron emission tomography–computed tomography, FDG: fludeoxyglucose-18

A PET-CT scan was also performed to characterize the hepatic tumor and to search for active metastatic lesions (Figure 5).

The patient’s blood test revealed the following results: alpha-fetoprotein (AFP) level of 179,621.2 ng/mL (normal value <6 ng/mL), HBsAg of 1,989 IU/mL (normal value: negative), and HBV DNA of 1,190.36 IU/mL (normal value: undetectable). These findings led to the patient being referred to the infectious diseases department, where anti-HBV treatment with Entecavir was initiated.

**Figure 6:**
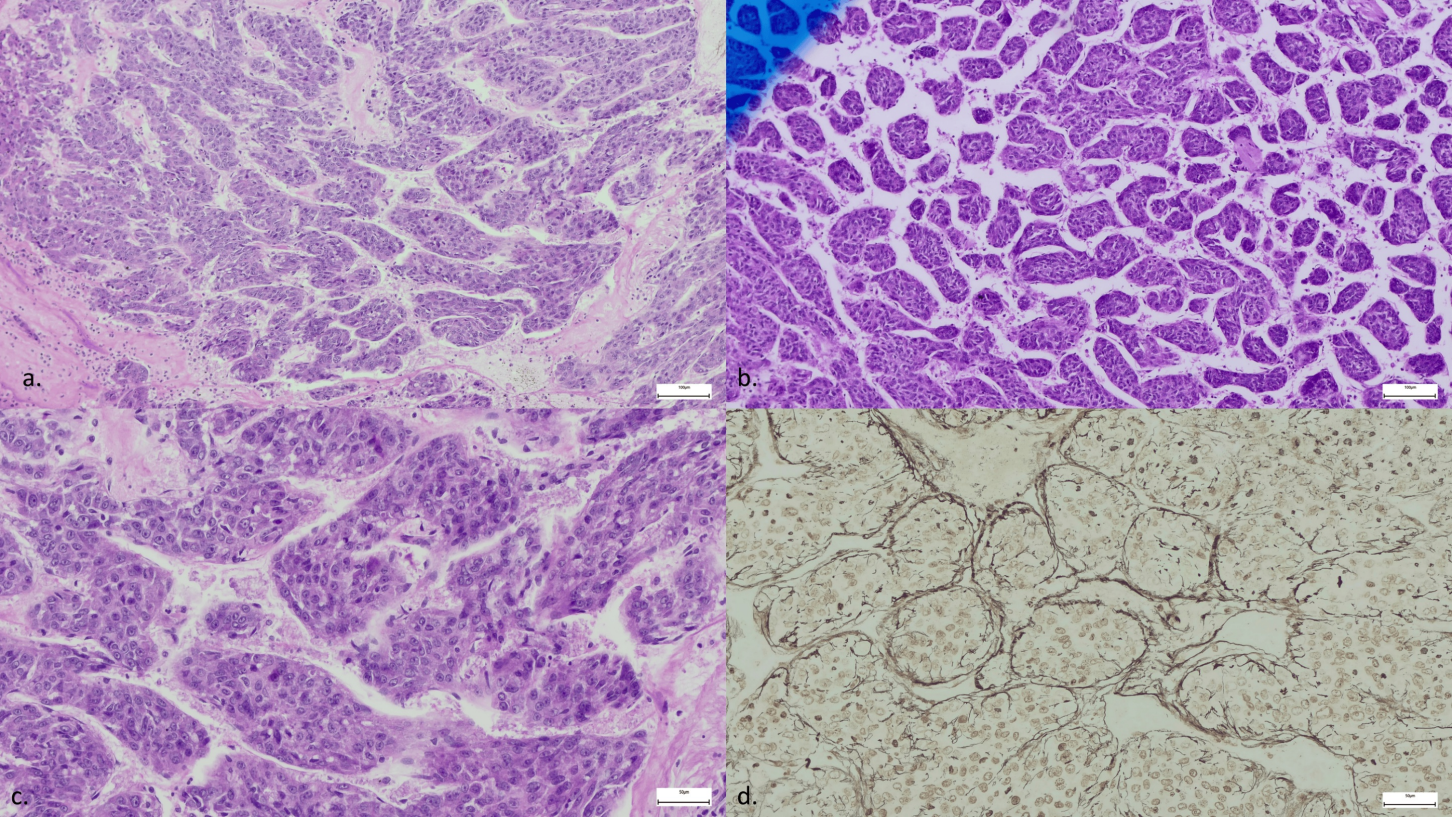
Histological features of MTM-HCC a, b: Histologic sections show thickened cords composed of polygonal tumor cells with eosinophilic cytoplasm and macrotrabeculae with a thickness of ≥6–10 cells. c: Tumor cells in the MTM-HCC exhibit eosinophilic cytoplasm, an increased nuclear-to-cytoplasmic ratio, and prominent nucleoli. d: Gomori stain: reticulin fibers focally encircle the thickened cords of tumor cells. MTM-HCC: macrotrabecular-massive hepatocellular carcinoma

An ultrasound-guided liver biopsy was performed, and histopathological examination identified a tumor with a macrotrabecular growth pattern, characterized by trabeculae thicker than 10 tumor hepatocytes and solid areas occupying more than 50% of the tumor cells (Figure 6). The final diagnosis was moderately differentiated hepatocellular carcinoma (G2) associated with the macrotrabecular subtype.

The patient was admitted to our oncology department to initiate treatment. The tumor was classified as T4N0M1 with venous invasion, peritoneal metastasis (Figure 2a), and pulmonary metastasis (Figure 2b).

The patient consented to the recommended treatment plan. In accordance with the Food and Drug Administration's current guidelines, the patient received C1 atezolizumab at a dose of 1200 mg and bevacizumab at 15 mg/kg with no acute adverse reactions. He continued the same immunotherapy regimen every three weeks.

After four treatment sessions, tumor evolution was revealed during a physical examination of a distended abdomen. A thoracic-abdominal-pelvic CT scan was performed, confirming disease progression. The patient died less than two months after his last consultation.

## Discussion

HBV and the pathogenesis of MTM-HCC

HBV infection is the most common risk factor for HCC. Studies suggest that 50 to 80% of HCCs are associated with HBV [10]. We have to highlight that HBV infection is a common risk factor for MTM-HCC [7,9]. The amount of HBV DNA is significantly correlated to a poor HCC prognosis [11]. It is important to note that HBV can lead to the development of HCC without causing liver cirrhosis, affecting up to 0.6% of patients infected with HBV [12].

The WHO classification of tumors recognizes eight subtypes of HCC: steatohepatitic, clear cell, scirrhous, chromophobe, fibrolamellar carcinoma, neutrophil-rich, lymphocyte-rich, and mactrotrabecular massive [5]. This last subtype represents 5% of all HCC subtypes. From a histological point of view, this subtype often presents with frequent vascular invasion and poor differentiation [7,9,13].

From a genetic perspective, MTM-HCC is frequently associated with TP53 mutations and FGF19 amplification [7,9,13]. Additionally, studies report a higher expression of PD-L1 and CMTM6 in MTM-HCC [8], with CMTM6 and PD-L1 appearing to be independently associated with poor prognosis.

In HCC, biomarkers indicative of poor prognosis generally include AFP-L3, DCP, and osteopontin [14]. Specifically, in MTM-HCC, elevated serum AFP levels are commonly observed, with studies [7,9,13] describing high AFP levels as an indicator of poor prognosis. MTM-HCC is also associated with a high frequency of metastasis [7,9,13]. In our case, the patient presented with AFP levels exceeding 50,000 UI/mL, consistent with a poor prognosis.

Diagnostic challenges

For MTM-HCC specifically, various approaches can be used to assess prognosis. MRI, for instance, may reveal distinctive features of this subtype. For example, one study proposed new criteria to characterize MTM-HCC lesions, termed the "MRIC-2" criteria. These criteria include a hypovascular component of ≥50% and two or more ancillary findings (intratumoral artery, arterial phase peritumoral enhancement, and non-smooth tumor margins). This classification was significantly associated with MTM-HCC and indicates a poor prognosis [15].

Sessa et al. suggest that artificial intelligence (AI) may become crucial for diagnosing and predicting MTM-HCC prognosis [16]. For diagnosis and prognosis, AI can integrate biomarkers, biopsy results, and clinical data to estimate outcomes.

Therapeutic strategies and prognostic outlook

HCC treatment efficacy is very dependent on the stage of the disease at the time of the diagnosis. Unfortunately, most of the time, the diagnosis occurs at the intermediate or advanced stage. Regarding these cases, the FDA guidelines recommend using immune checkpoint inhibitors [17]. Nevertheless, the efficacy of treatments also depends on the subtypes of HCC.

Current FDA guidelines recommend the use of an atezolizumab-bevacizumab combination for patients with unresectable locally advanced or metastatic HCC who have not received prior systemic treatment [18]. However, the FDA does not specify a treatment protocol specifically for MTM-HCC. The methods used for the IMbrave150 trial, which led to the approval of this treatment, did not include differentiation in treatment outcomes based on HCC subtypes [19].

Current guidelines generally advise against routine biopsies for diagnosing HCC in order to minimize patient discomfort. However, this approach can limit the ability to accurately identify HCC subtypes, which is crucial for determining prognosis and treatment strategies, as different subtypes are associated with varying mortality rates. Several radiology studies have described imaging features specific to MTM-HCC [15,20,21], which could aid in diagnosing this subtype without the need for a biopsy.

The findings of Liu et al. suggest that the treatment of MTM-HCC may differ from standard HCC protocols [8]. Another study, based on preclinical findings, proposes that angiogenesis inhibitors, such as VEGFA-targeting drugs like bevacizumab and ANGPT2 inhibitors, both already in clinical use, may enhance antitumor immunity when combined with PD-1 checkpoint blockade [22].

Lessons from this case

This case is significant due to its rarity, illustrating a clinically distinctive presentation of MTM-HCC. The comprehensive description, along with MRI and histology images, provides valuable references that may assist clinicians in identifying similar cases in the future. However, the lack of follow-up limits our understanding of the patient’s response to alternative treatments and restricts insights into potential long-term outcomes.

While the clinical symptoms of HCC, in general, are well-documented, such as abdominal pain, weight loss, fatigue, jaundice, ascites, and palpable abdominal masses, symptoms distinguishing the MTM subtype remain underreported. Our case did not exhibit any specific signs beyond those commonly documented for HCC in general, apart from the advanced stage at the time of the initial consultation.

## Conclusions

This case contributes to the limited literature on MTM-HCC, highlighting the need for tailored therapeutic approaches. The notably young age of our patient underscores the importance of early diagnosis and clinical vigilance, even in patients with mild symptoms. Further research, including multicenter collaborations, is critical to explore predictive genetic factors and molecular pathways involved in this aggressive subtype. These efforts could pave the way for innovative therapeutic strategies, such as combining immunotherapy with angiogenesis inhibitors, to improve outcomes for patients with MTM-HCC.
